# Modeling the Average and Instantaneous Friction Coefficient of a Disc Brake on the Basis of Bench Tests

**DOI:** 10.3390/ma14164766

**Published:** 2021-08-23

**Authors:** Wojciech Sawczuk, Armando Miguel Rilo Cañás, Dariusz Ulbrich, Jakub Kowalczyk

**Affiliations:** 1 Faculty of Transport and Civil Engineering, Institute of Transport, 60-965 Poznan, Poland; wojciech.sawczuk@put.poznan.pl; 2 DB Systemtechnik GmbH, 14774 Berlin, Germany; armando-miguel.rilo-canas@deutschebahn.com; 3 Faculty of Transport and Civil Engineering, Institute of Machines and Motor Vehicles, 60-965 Poznan, Poland; jakub.kowalczyk@put.poznan.pl

**Keywords:** railway disc brake, friction coefficient, multiple regression

## Abstract

This article presents the results of tests conducted on the average and instantaneous friction coefficients of railway vehicle disc brakes. The tests were carried out independently of various states of wear on the friction linings and the brake disc. The requirements of the International Union of Railways (UIC) regarding the approval of brake linings for use were taken into account. Based on many years of research using a brake bench to test railway disc brakes, the authors developed multiple regression models for the average friction coefficient and fluctuations (tolerances) in the instantaneous friction coefficient and achieved 870 results. The models proposed three types of variables: the input braking parameters (speed, pressure, and mass to be braked), operational parameters (the wear on the friction linings and the brake disc), and design parameters (perforations in the form of holes on the disc surface). The above two models were validated on the basis of 384 brakes, and in subsequent stages a further evaluation was performed. The coefficients were determined to be, respectively, 0.99 for the model of the average friction coefficient and 0.71 for the model of tolerance (fluctuations) of the instantaneous friction coefficient.

## 1. Introduction

Due to the increasing speed of passenger and freight trains, the friction disc brake has become the basic braking device. The main advantage is the constant value of the friction coefficient in the entire braking speed range compared to the classic block brake. Despite the many advantages of this braking system, it is difficult to control the wear on the disc-lining friction pair because the discs are mounted on the axle between the wheels of a wheelset.

Manufacturers of braking systems now have to increase the effectiveness of brakes on newly developed rail vehicles because railways are being modernized and expanded to accommodate trains at speeds up to 350 km/h.

It is possible to stop a train in any condition, even when there is ice on the contact line, an obstruction on the track, or something in the brake causing a reduction in the friction coefficient [1,2]. For passengers on high-speed trains, fluctuations in the friction coefficient translate into changes in braking deceleration, which can be unpleasant.

The instantaneous friction coefficient may drop significantly if there is something interfering with the contact between the lining and the brake disc. This reduction, especially when braking at high speeds (above 160 km/h), may be influenced by a frictional–mechanical phenomenon or heat. The result is the formation of a “third” layer in the contact between the lining and the disc that increases the slip of the lining against the brake disc and causes wear on the friction pair as described in [3,4]. Because it is difficult to remove worn friction material, braking times become longer.

Whatever interferes with the friction pair of the brake makes subsequent braking non-repeatable under the same initial conditions: the temperature of the friction pair, the pressure of the lining against the disc, or the mass (per disc) that has to be braked. To maintain the desired braking parameters, such as a constant deceleration when stopping or constant power for slope braking, a control system was introduced to change the pressure value in relation to the speed. This assumption and the manufacturers’ response are focused on automation and, consequently, on braking regulation, which allows for continuous monitoring by a number of sensors that send signals to actuators that maintain the braking parameters. Moreover, such an approach to controlling the brake friction systems entails cooperation with an electrodynamic brake. In the case of locomotives and multiple electric units, the traction motors during braking generate additional resistance in the form of torque Several brake systems work with a brake controller (called blending or “brake mixing”) [1,5,6] in which the control and monitoring system is responsible for stopping the vehicle at the assumed initial conditions regardless of environmental condition or what occurs in the friction pair.

It should be noted that this is a systems approach to braking because if the electrodynamic brake should lose effectiveness of something happens at the contact between the lining and the brake disc, the control system forces the friction brake to work more intensively. If it becomes overused in relation to the electrodynamic brake or has an operational malfunction, damage will occur in the form of accelerated wear on the friction material, non-linear wear on the brake disc, or cracks in the disc surface. These cracks are the result of what is referred to in the literature as “hot spots”, local overheating of the brake disc because of an uneven distribution of pressure from the lining against the disc [7,8,9,10,11,12].

In ref. [13], on the basis of numerical analyses and subsequent bench tests, it was shown that perforations in the friction surface of the disc improved the frictional and thermal characteristics for some types of braking, especially those with a high thermal load.

All tests in friction brakes are preceded by analyses in relation to dry friction. This applies to static and kinetic friction and is presented in a number of works [14,15,16,17,18,19,20,21]. The operating range of a disc brake is very complex, a function of speed and load and the transition states between rest and kinetic friction. A large number of variable parameters make friction modeling of a braking system difficult, requiring a significant extension of the model and a longer calculation time.

Bench tests are often preceded by laboratory tests of samples of the disc friction material and linings as well as numerical calculations carried out in programs such as SolidWorks, Ansys, or Abaqus [22,23,24]. Then the temperature distribution on the brake disc can be assessed [25,26,27]. For this, many researchers explain and model the hot spots on the disc surfaces [28,29]. Another issue they raise is fatigue cracking [3,28,30]. Cracks appear on the friction surface of a disc in the form of a mesh of microcracks [28,29,30,31,32] caused by the braking heating–cooling cycle. To avoid surface cracks, many research centers are conducting research into new materials for brake discs [33,34,35,36,37].

Vibro-acoustics generated by friction brakes is a separate issue that has been analyzed, and many studies have been written explaining their causes or attempting to model them [38,39].

The aim of this article is to present multiple regression models to describe the changes in the friction coefficient of the railway vehicle disc brake, taking into account the braking, disc design, and operational parameters of wear on the friction pair elements.

It should be emphasized that current regulations for approving brake system elements come down to a positive test result on a certified brake stand but only for new (not worn) brake discs and lining; and no check is made of the frictional characteristics of partially or completely worn elements.

## 2. Materials and Methods

**Figure 1:**
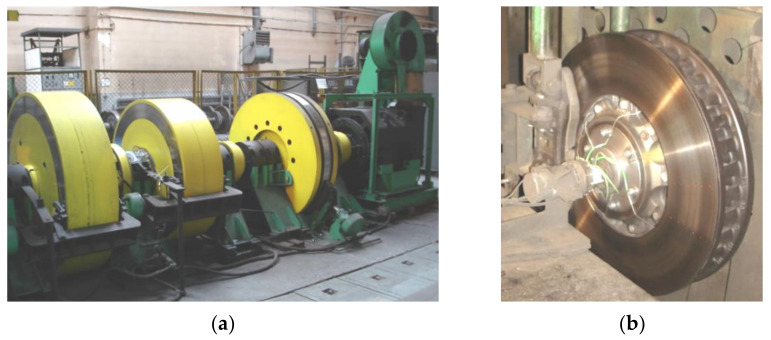
Brake bench for testing railway disc brakes: (**a**) drive part of the brake stand with rotating masses, (**b**) brake disc type 610 × 110 mounted on a brake bench.

During the tests, the input parameters (the condition of the braking system) were changed in a specific manner, and their influence on changes to the output parameters, such as the braking start speed, brake pad pressure, braking mass or friction lining wear, was observed. Tribological tests were carried out on the inertial brake stand (Figure 1). The test bench allows for the performance of railway block-brake and disc-brake tests and reflecting the actual conditions that occur during braking. Bench tests on certified stands, deliver the appropriate characteristics of instantaneous and average friction coefficients.

The tests in the first stage covered two ventilated brake discs with dimensions of Ø610 × 110 made of gray cast iron. The new disc was 110 mm thick and weighed 116 kg; the worn disc was 105 mm thick and weighed 111.5 kg. Both were prepared in accordance with standard procedure [40], and organic friction linings were used. Based on the literature, disc brakes, including those with perforations on the friction surface [41], a new friction ring surface with holes drilled along an Archimedes spiral (the third disc in the second stage of bench tests) was developed in conjunction with the authors’ considerations for the tests. One turn of the spiral improved the frictional characteristics of the brake and did not significantly increase wear on the lining. The Archimedes spiral in polar coordinates can be written [42] as:

(1) $$R\left(\varphi \right)=\frac{\left(k_{s}\times \varphi +\Delta k_{s}\right)}{2\pi }\times \left(\varphi +\Delta \varphi \right)$$

where φ is the variable of the angle expressed in radians; R(φ), the function of the dependence of the radius on the angle φ; k_s_, the parameter specifying the distance increment between the individual turns of the Archimedean spiral; Δk_s_, the parameter moving the leading radius of the function by a given value; and Δφ, the parameter moving the starting angle of the Archimedean spiral.

**Figure 2:**
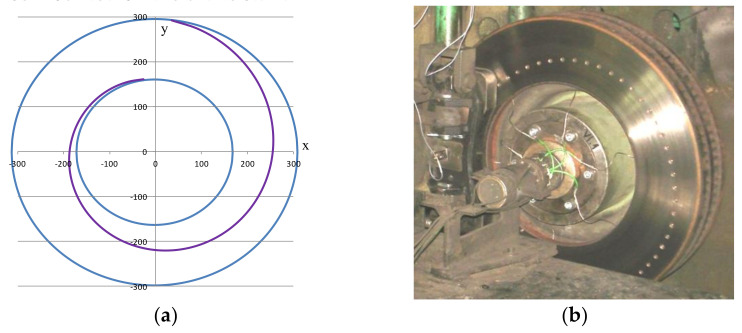
View of the brake disc: (**a**) diagram of the Archimedes spiral with one turn limited by the functions of a circle, (**b**) disc on the brake bench.

For research purposes, the holes were made on the brake disc along a spiral line written with the following system of parametric equations:

(2) $$\left\{\begin{array}{c} x\left(t\right)=A_{s}^{t}\times \cos\left(t\right)\\ y\left(t\right)=A_{s}^{t}\times \sin\left(t\right) \end{array}\right.\;t\in R,\;R\in \langle 52:76.2\rangle$$

where A_s_ is the Archimedes spiral parameter 1.103. Figure 2a shows a diagram of the spiral according to Equation (2) in the parametric coordinate system, taking into account the function of the circle for the external and internal radius of the brake disc. Figure 2b shows the disc mounted on the brake stand.

This solution to the brake disc profile with one turn of the Archimedes spiral was submitted to the Patent Office of the Republic of Poland [43].

The FR20H.2 friction linings were made of thermosetting resin, synthetic elastomer, metal, organic fibers, and friction modifiers [44]. In a few vehicles, such as a subway car, some multiple electric units and linings made of sintered metal were used. Papers [45,46] present the results of the bench tests of a railway disc brake with sintered friction linings.

**Figure 3:**
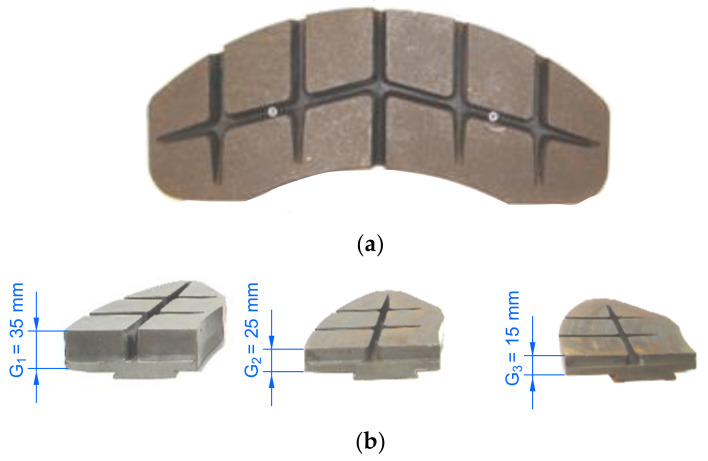
View of the friction linings used during bench tests: (**a**) view with visible expansion grooves, (**b**) side view with visible lining thickness.

In tests of the friction linings, it was important to obtain positive braking results at various speeds, pressures, and masses. In the case of cladding made of organic material, the average friction coefficient should be 0.35 [44]. Figure 3 shows the view of the organic friction linings used during the stand tests.

For bench tests, three sets of linings were used for each disc. The new lining kit was 35 mm thick, whereas the worn lining sets were 25 and 15 mm (Figure 3b).

The tests were carried out in accordance with the UIC 541-3 standard. To reflect the actual conditions that occur during the braking of passenger cars with a disc brake, the research program C (fast driving) was selected on the basis of Annex 01 to RP-0281 of the brake disc testing program developed by the Łukasiewicz Research Network, TABOR Institute of Rail Vehicles in Poznań, Poland. The variable (controlled) parameters during friction–mechanical tests are included in Table 1.

Before starting the tribological bench tests, a series of brakings was carried out on the lapping of friction linings. An additional 15 brakings were made for each set of friction linings to rub and arrange them to the brake disc. During the tests, the instantaneous tangential force F_t_ related to the braking radius r_h_ and the instantaneous pressure force on the brake disc F_b_ were simultaneously recorded. On this basis, the instantaneous friction factor µ_a_ was calculated [44] as

(3) $$\mu_{a}=\frac{F_{t}}{F_{b}}$$

where F_t_ is the instantaneous tangential force (kN), and F_b_ is the momentary pressure force of the linings to the disc (kN). Then, the average coefficient of friction µ_m_ was calculated as the integral determined from the instantaneous coefficient of friction along the braking distance s_2_ as shown in Equation (4) [44].

(4) $$\mu_{m}=\frac{1}{s_{2}}\int_{0}^{s_{2}}\mu_{a}\mathrm{ds}$$

where s_2_ is the braking distance (m) and µ_a_ is the instantaneous coefficient of friction.

During the tribological tests, 870 brakings were performed without taking into account those associated with the lapping of the friction linings. To validate the multiple regression model, another 384 were performed.

Changes in the friction coefficient, apart from the presented design, operational and initial parameters, are also influenced by vibrations occurring during braking. Temporary disengagements from the disc and re-pressing of the pads against the disc according to the most popular model of vibrations in brakes, i.e., the stick-slip model, are strongly dependent on the material properties of the friction pads. To a lesser extent than the material of the brake disc. This model relies on dynamic parameters such as mass, stiffness, and damping. Each friction material will have different m, k, and c parameters. These parameters, due to the vibration amplitude of the friction linings, will affect the braking time and, consequently, the braking distance. Overall, the researchers found that the vibroacoustic events were not fully established, but the most likely explanation for these phenomena was stick-slip movement that occurs in frictional coupling, the energy source of which is the change in the coefficient of friction as a function of velocity. The model of stick-slip phenomena is presented in [47,48].

## 3. Results

The purpose of the stand tests was to determine the instantaneous and average coefficient of friction in accordance with the relationships in Equations (3) and (4). Then, the results were checked against the requirements contained in [44]. 

**Figure 4:**
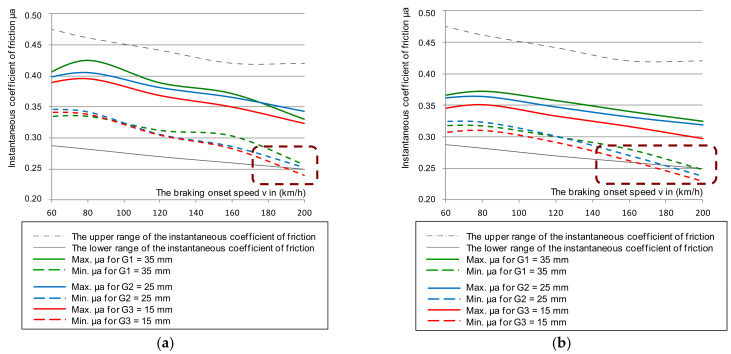
Characteristics of the instantaneous coefficient of friction μ_a_ on the braking initiation speed during braking with a pressure of 44 kN and mass per disc of 7.5 t: (**a**) for a new disc, (**b**) for a worn disc.

Some results of the tests of the instantaneous coefficient of friction for cladding thicknesses G_1_ = 35, G_2_ = 25, and G_3_ = 15 mm together with the smooth discs (new and worn without perforations) are shown in Figure 4, taking into account the upper and lower limits of the instantaneous coefficient of friction.

An analysis of changes in the instantaneous coefficient of friction presented in Figure 4 stated that in some braking combinations (i.e., the brake pad pressure on the disc and braking mass), the μ_a_ values were lower than the minimum required value of the instantaneous friction coefficient contained in [44]. This phenomenon especially occurred on a disc worn to a thickness of 105 mm and linings worn to a thickness of 15 mm. Other conditions of the test were a braking pressure of 44 kN and a braking mass of 7.5 t, which simulated the braking of a train with a maximum load at a speed of 200 km/h. For a new disc, only worn linings affected the instantaneous coefficient of friction for the lower μ_a_ tolerance limit at a braking speed of 200 km/h. However, it should be expected that at braking speeds above 200 km/h, the instantaneous coefficient of friction would drop below the required value.

**Figure 5:**
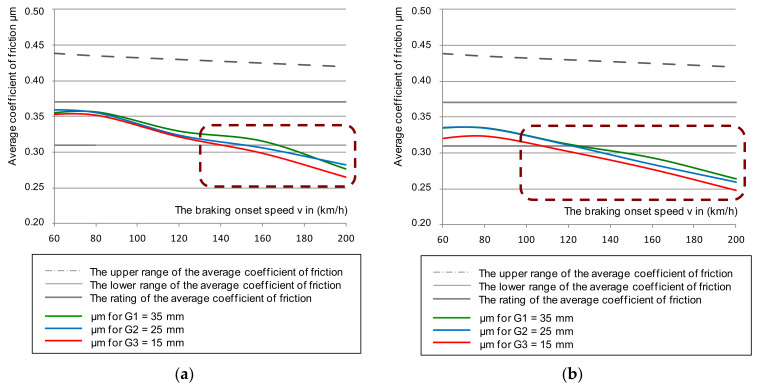
Characteristics of the average friction coefficient μ_m_ on the braking start speed with a pressure of 44 kN and mass per disc of 7.5 t: (**a**) for a new disc, (**b**) for a worn disc.

Based on Equation (4), the mean value of the friction coefficient was determined. The selected dependence of the average coefficient of friction for the same braking parameters as in the instantaneous coefficient test is shown in Figure 5. The results also referred to the upper and lower deviation from the mean friction coefficient.

Figure 5 shows the selected course of the average coefficient of friction as a function of braking speed (N = 44 kN and M = 5.7 t). However, based on the remaining changes during the tests, the lower deviation of the average coefficient of friction in all braking cases was exceeded for both the new and worn disc brakes, with all friction linings, new and worn.

Only in the case of braking with low pressure (28 kN) and braking mass (4.4 t) on a new disc with new brake pads up to the braking speed of 200 km/h, did the mean coefficient of friction not exceed its lower limit. Braking with high pressure (44 kN) and mass (7.5 t) on a new disc and worn linings caused a failure in the lower limit requirement of the average coefficient of friction. However, it should be noted that the tests did not use the most worn friction linings and brake discs. It was allowed to use, on the basis of the operation and maintenance documentation [49,50], a disc worn down to a thickness of 102 mm by cyclical turning, and for the friction linings to wear down to 5 mm on the basis of [51,52]. In the tests, a disc with a thickness of about 105 mm was used and the brake pads were worn down to a maximum thickness of 15 mm. After a series of 480 brakings, they were 7 mm thick.

During the bench tests, the influence of brake disc perforations on the instantaneous and average friction coefficients was also checked. A disc with holes drilled along the Archimedes spiral with one turn was used, and the results were related to the same disc with a classic (smooth) friction surface. The first testing stage with holes along the Archimedes spiral with one turn, was compared to the classic, smooth disc, to determine time-domain characteristics to make it possible to observe fluctuations in the instantaneous coefficient of friction throughout the braking.

**Figure 6:**
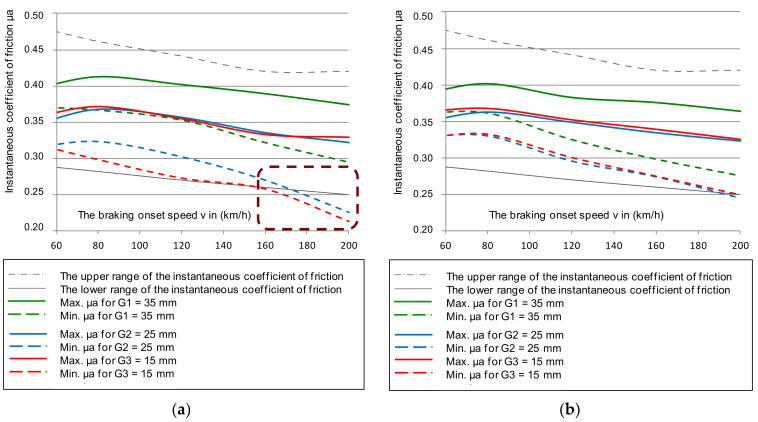
Characteristics of the instantaneous coefficient of friction μ_a_ on the braking initiation speed with a pressure of 36 kN, and mass per disc of 4.7 t: (**a**) for a classic disc, (**b**) for a disc perforated on the friction surface.

**Figure 7:**
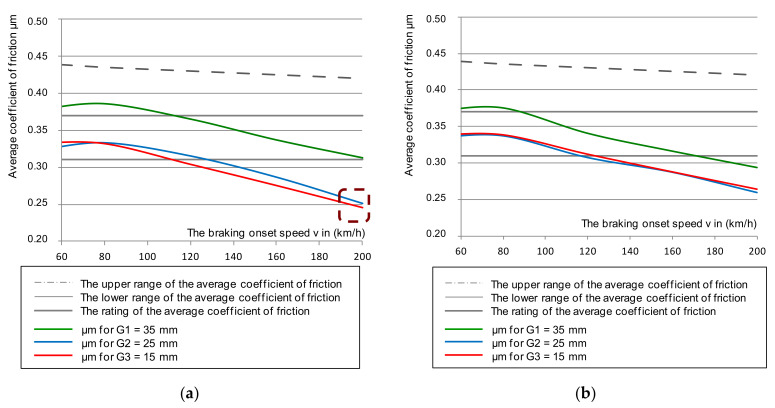
Characteristics of the average coefficient of friction μ_m_ on the braking initiation speed with a pressure of 36 kN and mass per disc of 4.7 t: (**a**) for a classic disc, (**b**) for a disc perforated on the friction surface.

Figure 6 shows the cumulative characteristics of μ_a_ (minimum and maximum from a given braking test) as a function of braking speed: 50–200 km/h for a brake pad pressure of 36 kN. On the other hand, in Figure 7, for the same pressure, the dependence of the average μ_m_ coefficient of friction from two discs (smooth without perforation, and on the disc with holes along the Archimedes spiral) was presented. In addition, the impact of friction-lining wear on changes to the friction coefficients was taken into account. This was important for the disc brake operation concerning fulfilment of the temporary tolerance and the average coefficient of friction imposed by UIC 541-3. During the test, new (lapped) 35 mm linings and linings worn down to a thickness of 25 and 15 mm were used.

Based on an analysis of the graphs in Figure 6 and Figure 7, changes in the instantaneous and average friction coefficients for a classic smooth disc and one with holes in a spiral line showed that changes in the range of μ_a_ and μ_m_ were smaller in speed function at the beginning of braking for a drilled disc than for one with a smooth friction surface. Even a change in the thickness of the friction linings in the disc brake did not significantly reduce the friction coefficient in the disc with holes.

**Figure 8:**
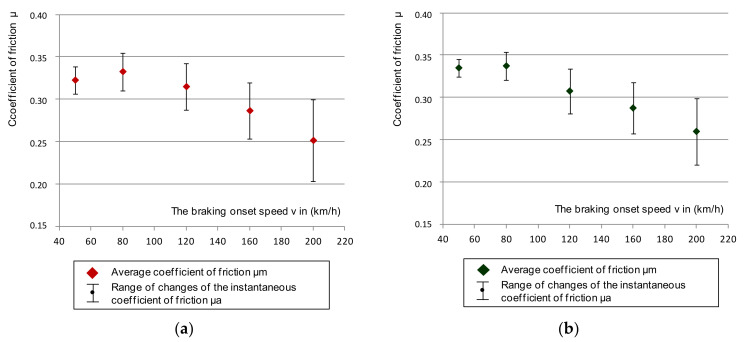
Characteristics of the friction coefficient (average and its spread) on the braking initiation speed with a pressure of 36 kN and mass per disc of 4.7 t: (**a**) for a classic disc, (**b**) for a disc perforated on the friction surface.

In Figure 8, the average values of the braking speed friction coefficient are marked with points, while the fluctuations of the instantaneous friction coefficient are marked as an error using the maximum and minimum values obtained during braking for both discs (smooth and perforated).

Table 2 shows the tolerance of the instantaneous coefficient of friction (μ_a_ and μ_m_) obtained during braking on a disc with a smooth (classic) friction surface and on a disc with holes in a spiral line. The tolerance of the friction coefficients as the difference between the maximum and minimum value was determined for all braking speeds from 50 to 200 km/h and for the thickness of the friction linings (35, 25, and 15 mm) but separately for the two friction lining pressures on the disc brake (25 and 36 kN).

Based on Figure 8 and Table 2, the perforations in the Archimedean spiral line caused smaller fluctuations in the average and instantaneous coefficient of friction in relation to the smooth disk. When braking with a pressure of 25 kN, fluctuations in both µ_a_ and µ_m_ for the perforated disc were approx. 1.6 times smaller than for the smooth disc, and 1.3 times lower for braking with a pressure of 36 kN.

**Figure 9:**
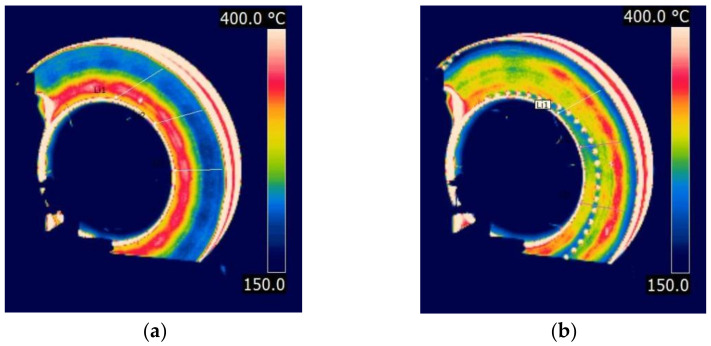
Temperature distribution on a disc at the moment braking ended from a speed of 200 km/h: (**a**) classic (smooth), (**b**) perforated on the friction surface.

Figure 9 shows thermal images of the temperature distribution on brake discs (smooth and with perforation) at the moment braking ended from a speed of 200 km/h.

Perforations on the friction surface improved the pressure distribution of the linings against the disc, which was confirmed by thermal images with temperature distribution. In the case of the classic smooth disc, the formation of hot spots at the inner radius of the disc was observed in the IR images. The perforations improved the removal of wear products formed when the friction linings made contact with the brake disc. Additional through holes improved heat transfer to the outside and reduced stress on the friction surface. The uneven distribution of the pressure of the linings against the disc affected stability and fluctuations in the instantaneous coefficient of friction.

## 4. Modeling of the Friction Coefficient

Based on the results of the instantaneous and average coefficients of friction, an attempt was made to model the values of μ_m_ and T_μa_ on the basis of braking, disc design, and operating parameters (wearing of the brake friction pair elements). The courses of the instantaneous coefficient of friction in the functioning of the braking time were particularly analyzed to determine the maximum and minimum values. Based on the result, it was possible to determine the tolerance (fluctuations) of the instantaneous coefficient of friction as the difference of its maximum and minimum value according to the relationship in Equation (5):

(5) $${T\mu }_{m\_\mathrm{bad}}{=\mu }_{a\;\max}\;-{\;\mu }_{a\;\min}$$

The modeling of both the average µ_m_ and the tolerance T_µa_ of the instantaneous coefficient of friction was preceded by checking the level of the linear relationship between individual variables such as the type of friction surface, wear on the brake disc and friction linings, speed, braking pressure on the disc, and braking mass. For the wear on the brake disc and friction linings, the thickness of the friction discs and linings were used as variables. Then, the Pearson’s linear correlation coefficient [53] was checked, in accordance with Equation (6), for the analyzed variables.

(6) $$r_{\mathrm{xy}}=\frac{\sum_{i=1}^{n}\left(x_{i}\;-\;\bar{x}\right)\left(y_{i}\;-\;\bar{y}\right)}{\sqrt{\sum_{i=1}^{n}{\left(x_{i}\;-\;\bar{x}\right)}^{2}}\sqrt{\sum_{i=1}^{n}{\left(y_{i}\;-\;\bar{y}\right)}^{2}}}$$

where $\bar{y}$, $\bar{x}$ are the mean values of the feature x and the feature y, and y_i_, x_i_ are the describing variables.

Table 3 and Table 4 show the correlation matrix (Pearson) for the variables of the model of the average coefficient of friction and the tolerance of the instantaneous coefficient of friction. Table 5 presents the values of the coefficients of variation for the average coefficient of friction and the tolerance of the instantaneous coefficient of friction. When analyzing the values of the correlation coefficient from Table 3, the conversion of the average friction coefficient was most influenced by the braking start speed (r = 0.79), and the brake pad pressure on the disc (r = 0.012), the type of friction surface (0.031), and deceleration mass. For the tolerance of the instantaneous coefficient of friction, the greatest influence was also the speed at the beginning of braking (0.409), and the least influence was the pressure of the disc linings (0.012) and the thickness of the friction linings (0.019).

**Figure 10:**
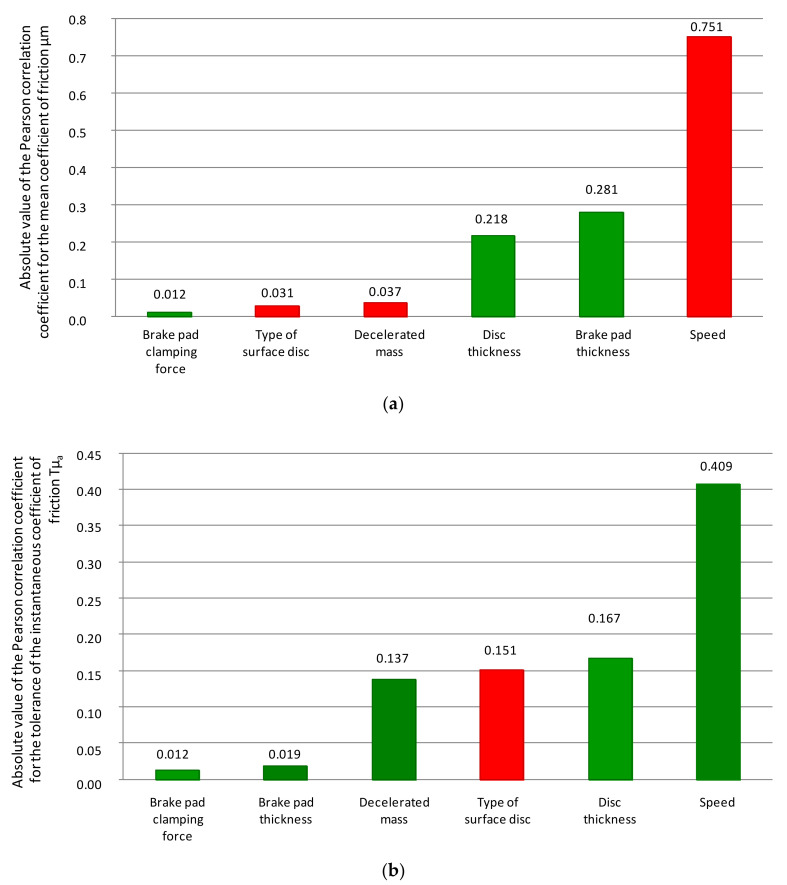
Distribution of the absolute value of the Pearson correlation coefficient for the variables of the regression model; (**a**) coefficient of friction and (**b**) tolerance (changes in the instantaneous coefficient of friction); red indicates an increase in the variable that caused a decrease in the value of µ_m_ or T_μa_; green indicates an increase in µ_m_ or T_μa_ with an increase in the value of the variable.

Figure 10 shows graphically the distribution of the Pearson correlation coefficient for the average model and the tolerance of the instantaneous coefficient of friction.

On the basis of the percentage coefficient of variation for the values of both populations, a low variability for µ_m_ and a moderate variability for T_µa_ were observed for the average coefficient of friction and tolerance of the instantaneous coefficient of friction.

It should be emphasized that, for fluctuations in the instantaneous coefficient of friction (also understood as the difference between the maximum and minimum values), the greatest influence was the braking start speed, wear on the brake disc, and the type of disc friction surface. The linear correlation coefficients were 41, 16, and 15%, respectively. This is important because it affected the constant value of braking deceleration throughout the entire braking time range and the comfort of passengers during braking.

A multiple regression model was used to describe the variability of the mean coefficient of friction and the tolerance of the instantaneous coefficient of friction. It is a method in which the value of a random variable Y depends on the k-th independent features (X1, X2, … Xk). On the basis of a given sample of the test results [54], the determination of invariable parameters α0, α1, … αk was performed using the least squares method. To determine the mean value of the coefficient of friction and the tolerance of the instantaneous coefficient of friction (changes in the braking time), the following relationships were proposed:

(7) $$\mu_{m}=\alpha_{1}A_{D}+\alpha_{2}G_{T}+\alpha_{3}G_{o}+\alpha_{4}v_{o}^{2}+\alpha_{5}v_{o}+\alpha_{6}N+\alpha_{7}M+\alpha_{0}$$

(8) $$T_{\mu m}=\beta_{1}A_{D}+\beta_{2}G_{T}+\beta_{3}G_{o}+\beta_{4}v_{o}^{2}+\beta_{5}v_{o}+\beta_{6}N+\beta_{7}M+\beta_{0}$$

The variables A_D_, G_T_, G_O_, v_o_, N, M with their values are presented in Table 1.

For each characteristic µ_a_ = f (t_h_), where t_h_ is the time of a braking from v_max_ to v = 0 (depending on the design and operational parameters of the friction pair and the input parameters of the braking process) the extreme value of the instantaneous friction coefficient after the braking time was determined. The maximum and local minimum of each function of the instantaneous friction coefficient were identified in accordance with the following assumptions:

(a) The function µ_a_(t_h_) at the point tn∈Df had a local maximum equal to µ_a_max_(t_n_) if and only if there was an environment U of the point tn such that for each
  t_n_∈U∩D_f_, D_f_ = R/{0}, R∈(0; t_h_> and t ≠ t_h_ there was an inequality
  µ_a_(t_h_) < µ_a_max_(t_n_)
(b) The function µ_a_(t_h_) had a local minimum at t_n_∈D_f_ equal to µ_a_min_(t_n_) if and only if there was an environment U of point tn such that for each:
  t_n_∈U∩D_f_, D_f_ = R/{0}, R∈(0; t_h_> and t ≠ t_h_ there was an inequality
  µ_a_(t_h_) > µ_a_min_(t_h_)

The calculated parameters of the multiple regression (Table 6) for models (7) and (8) were obtained with the coefficient of determination R^2^ = 0.78 for µ_m_ and 0.74 for T_µa_, in accordance with the relationship in [53]:

(9) $$R^{2}=\frac{\sum_{i=1}^{n}\left({\hat{y}}_{i}\;-\;\bar{y}\right)}{\sum_{i=1}^{n}{\left(y_{i}\;-\;\bar{y}\right)}^{2}}$$

where ${\hat{y}}_{i}$ is the theoretical value of the dependent variable (based on the model); $\bar{y}$ is the mean value of the y feature (dependent variable); and y_i_ is the describing variable (actual value).

For the highest values of the Pearson correlation coefficient, the models written with Equation (7) and (8) were simplified. For the model of the average coefficient of friction, the influence of several variables was eliminated, i.e., the pressure of the pads on the disc (N), the type of disc friction surface (AD), and the mass per disc (M). Within the tolerance of the instantaneous coefficient of friction, the model was simplified by eliminating the influence of the pressure N, the thickness of the friction linings (G_o_), and mass (per disc) to decelerate M. To determine the average value of the friction coefficient along with its tolerance (changes in the instantaneous coefficient of friction during braking), a simplified relationship was proposed:

(10) $$\mu_{m}=\alpha_{1}G_{T}+\alpha_{2}G_{o}+\alpha_{3}v_{o}+\alpha_{4}v_{o}^{2}$$

(11) $$T_{\mu a}=\beta_{1}A_{D}+\beta_{2}G_{T}+\beta_{3}G_{o}+\beta_{4}v_{o}^{2}+\beta_{0}$$

The calculated parameters of the multiple regression for models (10) and (11) with the coefficient of determination R^2^ = 0.99 for µ_m_ and 0.71 for T_µa_ are summarized in Table 7.

Both the models described by the dependencies (7) and (8), as well as the simplified models (10) and (11), were verified for their α_0-7_ and β_0-7_ coefficients, as shown in Table 8 and Table 9.

The Student’s *t*-distribution was used to test the hypotheses concerning the significance of individual regression coefficients. If the significance of F was lower than the assumed significance level α (α = 0.05), then there was a linear relationship between the dependent variable and all explanatory variables included in the models. In the case of models with relations (7) and (8), the coefficients α_6_, α_7_, and α_o_ for the µ_m_ model and the β_6_ coefficients for the T_µa_ model have *F* values above 0.05, which proved the need to eliminate them. This justified the need to simplify the models to the dependencies (10) and (11). Re-verification of the coefficients of the regression function together with the adjustment R^2^ (Table 9) for both models of the average coefficient of friction µ_m_ and the tolerance of the instantaneous coefficient of friction T_µa_ proved that all the coefficients had a significance function *F* below the value of 0.05. In order to determine the maximum and minimum value of the friction coefficient, it was possible to use the relationship:

(12) $$\mu_{\max}{=\mu }_{m}+\frac{1}{2}{T\mu }_{a}$$

(13) $$\mu_{\min}{=\mu }_{m}\;-\;\frac{1}{2}{T\mu }_{a}$$

The µ_max_ and µ_min_ models described by the relations (12) and (13) are based on the values of the average and tolerance of the instantaneous coefficient of friction.

## 5. Verification and Validation of the Model of the Variability of the Friction Coefficient

**Figure 11:**
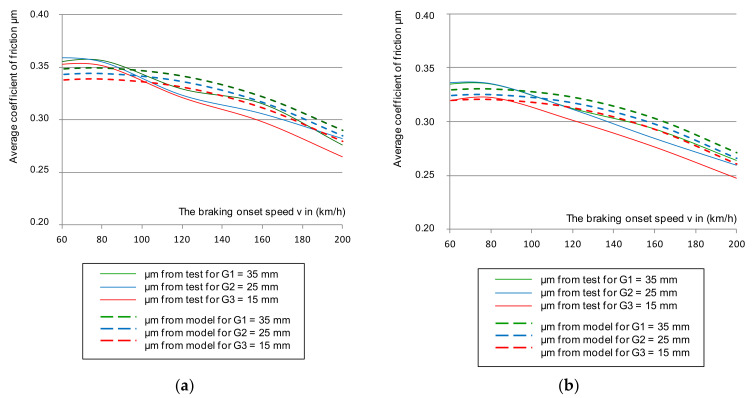
The μ_m_ changes from the tests with the multiple regression model during braking with N = 44 kN, M = 7.5 t for: (**a**) new disc, (**b**) worn disc.

The next stage was to verify the model of the average friction coefficient described by the Equation (10). Figure 11 shows the verification of a simplified regression model for a sample combination of braking with a pressure of 44 kN and a mass per disc of 7.5 t for a new and worn disc.

**Figure 12:**
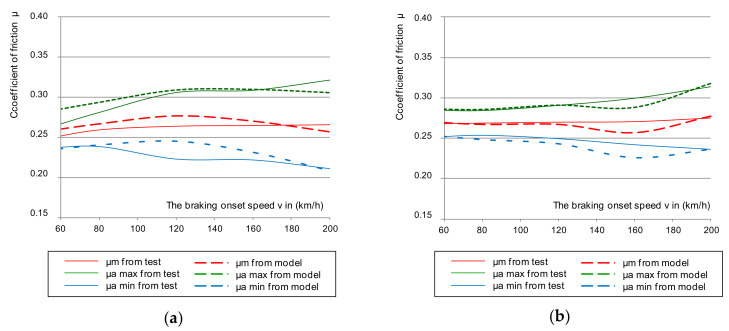
The changes of μ_m_ from the tests with multiple regression model during braking on the G_1_ lining with N = 25 kN, M = 5.7 t for: (**a**) smooth disc, (**b**) perforated disc.

**Figure 13:**
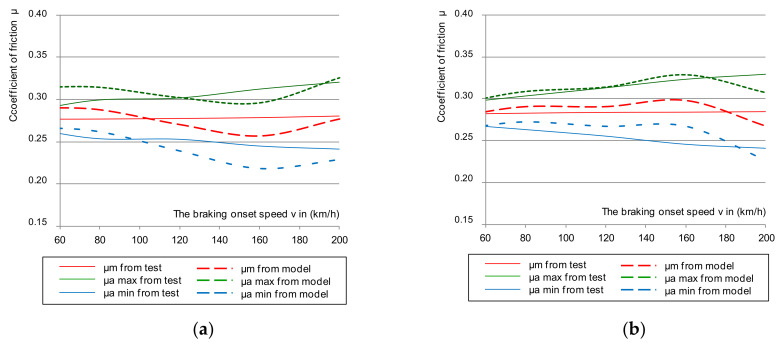
The changes in μ_m_ from the tests with multiple regression model during braking on the G_2_ lining with N = 25 kN, M = 5.7 t for (**a**) smooth disc and (**b**) perforated disc.

**Figure 14:**
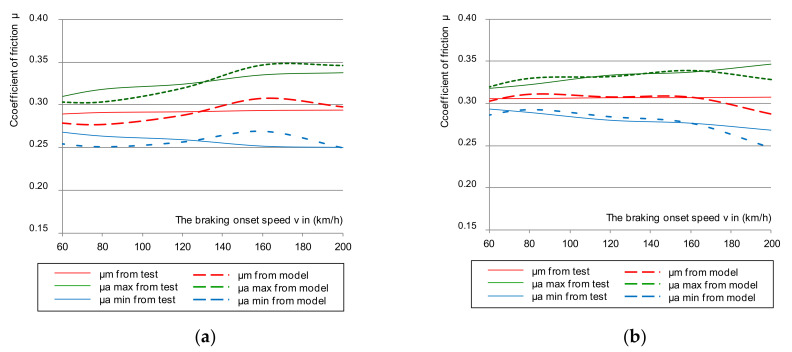
The changes of μ_m_ from the tests with multiple regression model during braking on the G_3_ lining with N = 25 kN, M = 5.7 t for: (**a**) smooth disc, (**b**) perforated disc.

On the other hand, Figure 12, Figure 13 and Figure 14 show the verification of the regression model in accordance with the relationships (11–13) in relation to the results of the average friction coefficient and the instantaneous coefficient of friction in the range of the maximum and minimum values obtained during braking for smooth and perforated discs. The verification of the regression model for both was carried out for three friction linings: new (35 mm) and two worn down to a thickness of 25 and 15 mm.

**Figure 15:**
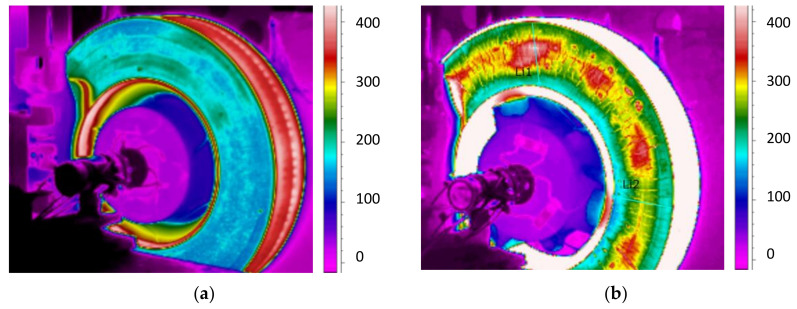
Thermal image of the brake disc, type: (**a**) 590 × 110 (new disc), (**b**) 640 × 110 (worn disc).

**Figure 16:**
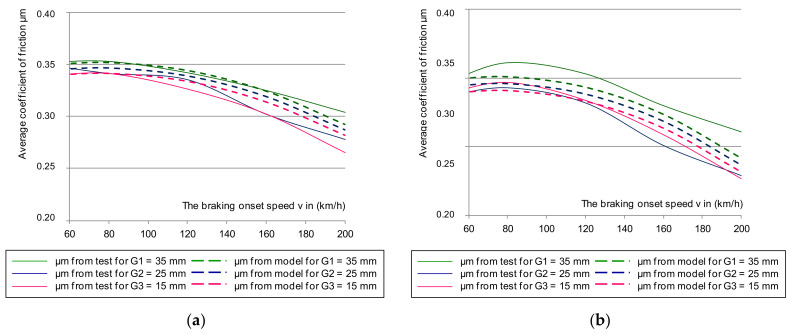
The changes of μ_m_ from the tests with multiple regression model during braking on a new disc type 590 × 110 z: (**a**) N = 25 kN and M = 5.7 t, (**b**) N = 36 kN and M = 5.7 t.

**Figure 17:**
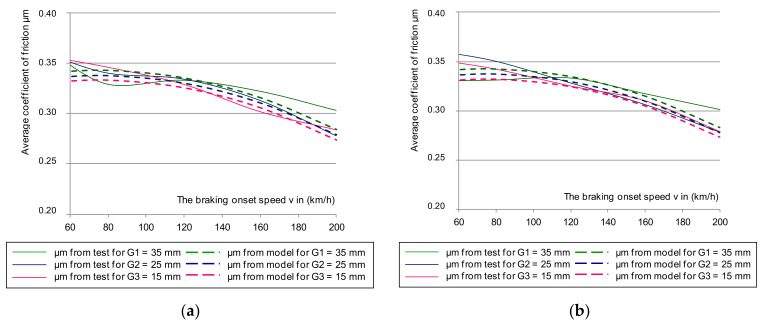
The changes of μ_m_ from the tests with multiple regression model during braking on a worn disc type 640 × 110 z: (**a**) N = 28 kN and M = 6.7 t, (**b**) N = 40 kN and M = 6.7 t.

Based on the analysis of Figure 12, Figure 13 and Figure 14, a satisfactory fit of the average model and the tolerance of the instantaneous friction coefficient to the values obtained from the stand tests was found. To verify the proposed model for estimating the value of the average coefficient of friction, the model from Equation (10) was also validated on two brake discs (new and regenerated) and friction linings made of organic material. The thermal images of the brake discs are shown in Figure 15. Microcracks were visible on the regenerated disc by a turning process from 110 to 108 mm. Figure 16 and Figure 17 show the validation of the regression model in accordance with Equation (10) in relation to the test results of the average friction coefficient obtained on the brake bench.

Then, the relative percentage error [55] of the multiple regression model of the average coefficient of friction related to the results of the test stand was determined. Due to the large sample (>30) on the basis of the inequality k ≤ 5log × n_p_, the number of classes (10) was established to present the distribution of the relative error [53]. Then, the maximum and minimum values of the variable (x_max_ = 9.8, x_min_ = 0.009) were determined, and the range of data was calculated (9.79). The largest relative error was 2%, resulting from the multiple regression model not fitting the test results. This error value occurred 44 times out of 120 observations, and an error of up to 5% occurred 88 times.

Next, the relative percentage error between the average coefficient of friction multiple regression model and the test results of a new brake disc (590 × 110 type) was determined. Due to the large sample (149 brakes), the number of classes k_lp_ = 10 was established to determine the relative percentage error distribution. Based on the relative error data, the variable’s maximum and minimum values (x_max_ = 13.4; x_min_ = 0.03) were determined, which made it possible to calculate the data range of 13.37.

The highest number of errors was relative error of up to 4%, resulting from mismatches of the multiple regression model to the test results, occurred 60 times out of 146 observations. In addition, for the regenerated disc, the relative percentage error of the fit of the average coefficient of friction multiple regression model to the results of the 640 × 110 brake disc was determined. For 237 decelerations with different pressures and masses, the number of classes k_lp_ = 11 was set to determine the percentage error distribution. Based on the relative error data, the maximum and minimum values (x_max_ = 14.6; x_min_ = 0.05) of the variable were determined, which made it possible to calculate the data range of 14.55. After the analysis, the relative error up to 7% was the highest number, which occurred 188 times out of 237 observations. From the distribution of the relative percentage error for all tested brake discs with friction linings worn to different degrees, it can be stated that the proposed model of the average friction coefficient satisfactorily reflected the actual values obtained from the tests. 

Verification of the average coefficient of friction model depended on the braking, operational and design parameters, and the relative error in the largest number of brakes was 2%. However, validation of the model on the new and worn disc gave a relative percentage error of 4 and 7%. It should be noted that the research and modeling of the average coefficient of friction µ_m_ were also presented in [56]. This article takes into account another variable from the group of design parameters; that is, disc perforation in the form of holes on the friction surface and its impact on frictional characteristics. Additionally, a model of the tolerance of the instantaneous coefficient of friction T_μa_ was proposed, which allowed us to determine its minimum and maximum values. Multiple regression models were developed on 870 brake applications with different combinations of input, operational, and design parameters.

## 6. Conclusions

Based on the research and analysis, the following conclusions can be drawn: 

The bench tests of the railway friction disc brake showed that, for some braking conditions and degree of wear on the brake components, the friction coefficient dropped and did not meet the requirements of the UIC 541-3 card. 

Changes in the average coefficient of friction of a disc brake μ_m_ were mostly influenced by the braking start speed and wear on the friction lining and brake disc. 

The input parameters of the braking start speed, brake disc wear, and type of disc friction surface (with or without perforations) had the greatest impact on the tolerance of the instantaneous coefficient of friction (T_μa_). 

A good fit was obtained between the models (average coefficient of friction and fluctuations of the instantaneous coefficient of friction) and the test results.

The relative percentage error during validation tests was in the 2–7% range.

The model designated in the article for µ_m_ and T_µa_ will be very helpful in designing brake systems for freight and passenger rail vehicles. On their basis, it will be possible to determine other braking parameters such as distance.

After complex and multi-stage tests on the abrasive friction in a disc brake consisting of a friction pair (organic friction linings and cast iron brake discs) it is justifiable to conduct similar tests on sintered linings. Organic linings (made of plastic) are used for vehicles designed for speeds up 200 km/h. In high-speed trains above this speed, sintered linings are needed to stop within the required braking distance.

## Figures and Tables

**Table 1:** Program of the bench tests.

No.	Variable Parameter	Symbol	Value	Unit
1	Type of disc friction surface	A_D_	0 (disc without perforation, smooth), 57 (disc with Archimedes spiral holes)	(–)
2	Condition of the brake disc	G_T_	110 (new), 105 (worn)	(mm)
3	Wear on the friction linings	G_o_	35 (new), 25 (partially worn out), 15 (worn out)	(mm)
4	Braking start speed	v_o_	50, 80, 120, 160, 200	(km/h)
5	The pressure of the linings to the disc	N	25, 28, 36, 44	(kN)
6	Braking mass per disc	M	4.4, 4.7, 7.5	(t)
Braking was made with a delay a = 0.9 (m/s^2^)				

**Table 2:** Friction coefficient tolerance.

Tolerance of the Coefficient of Friction at the Pressure of N = 25 kN		
–	Smooth disc	Disc with holes
Instantaneous coefficient of friction μ_a_	0.181	0.112
Average coefficient of friction μ_m_	0.117	0.076
Tolerance of the Coefficient of Friction at the Pressure of N = 36 kN		
–	Smooth disc	Disc with holes
Instantaneous coefficient of friction μ_a_	0.200	0.157
Average coefficient of friction μ_m_	0.140	0.115

**Table 3:** Correlation matrix for the average coefficient of friction µ_m_.

Variable	A_D_	G_T_	G_O_	v^2^	v	N	M	r_xy_ for µ_m_
Type of surface disc A_D_	1.0	0.316	0	0	0	−0.212	−0.059	−0.031
Disc thickness G_T_	0.316	1.0	0	0	0	−0.168	−0.046	0.218
Brake pad thickness G_O_	0	0	1.0	0	0	0	0	0.281
Speed v^2^	0	0	0	1.0	0.985	0	0	−0.785
Speed v	0	0	0	0.985	1.0	0	0	−0.751
Brake pad clamping force N	−0.212	−0.168	0	0	0	1.0	0.031	0.012
Decelerated mass M	−0.058	−0.046	0	0	0	0.031	1.0	−0.037
Correlation coefficient for µ_m_	−0.031	0.218	0.281	−0.785	−0.751	0.012	−0.037	1.0

**Table 4:** Correlation matrix for the tolerance of the instantaneous coefficient of friction T_μa_.

Variable	A_D_	G_T_	G_O_	v^2^	v	N	M	r_xy_ for T_µa_
Type of surface disc A_D_	1.0	0.316	0	−0.379	0.459	−0.212	−0.059	−0.151
Disc thickness G_T_	0.316	1.0	0	−0.299	0.363	−0.168	−0.046	0.167
Brake pad thickness G_O_	0	0	1.0	0	0	0	0	0.019
Speed v^2^	−0.379	−0.299	0	1.0	−0.431	0.201	0.056	0.406
Speed v	0.459	0.363	0	−0.431	1.0	−0.244	−0.067	0.409
Brake pad clamping force N	−0.212	−0.168	0	0.201	−0.244	1.0	0.031	0.012
Decelerated mass M	−0.059	−0.046	0	0.056	−0.067	0.031	1.0	0.137
Correlation coefficient for T_µm_	−0.151	0.167	0.019	0.406	0.409	0.012	0.137	1.0

**Table 5:** Values of the coefficients of variation for μ_m_ and T_μa_.

Coefficient of Variation w%		
For average coefficient of friction μ_m_	For fluctuations: instantaneous coefficient of friction Tμ_a_	<25%—low variability, (26–45%)—moderate variability, <46–100%)—strong variability, >100—very strong variability.
9.19	33.38	

**Table 6:** Multiple regression coefficients.

Coefficient	Value for µ_m_	Coefficient	Value for T_µa_
α_1_	−1.45 × 10^−4^	β_1_	−2.75 × 10^−5^
α_2_	3.15 × 10^−4^	β_2_	1.57 × 10^−3^
α_3_	9.99 × 10^−4^	β_3_	4.48 × 10^−5^
α_4_	−3.32 × 10^−6^	β_4_	9.37 × 10^−7^
α_5_	4.23 × 10^−4^	β_5_	1.32 × 10^−6^
α_6_	1.28 × 10^−4^	β_6_	8.07 × 10^−5^
α_7_	−7.35 × 10^−4^	β_7_	2.17 × 10^−3^
α_0_	−4.14 × 10^−2^	β_0_	−1.45 × 10^−2^
R^2^	0.78	R^2^	0.74

**Table 7:** Multiple regression coefficients of the simplified model of the average coefficient of friction and the tolerance of the instantaneous coefficient of friction.

Coefficient	Value for µ_m_	Coefficient	Value for T_µa_
α_1_	2.76 × 10^−3^	β_1_	−2.81 × 10^−4^
α_2_	10.0 × 10^−4^	β_2_	1.53 × 10^−3^
α_3_	4.26 × 10^−4^	β_3_	9.46 × 10^−7^
α_4_	−3.32 × 10^−6^	β_4_	1.31 × 10^−6^
α_0_	–	β_0_	−1.24 × 10^−1^

**Table 8:** Regression function coefficients with R^2^ fitting for µ_m_ and T_µa_ models before verification.

µ_m_			T_µa_		
Coefficient	Value	Value F *	Coefficient	Value	Value F *
α_1_	−1.45 × 10^−4^	0.0069	β_1_	−2.75 × 10^−5^	8.38 × 10^−9^
α_2_	3.15 × 10^−4^	3.81 × 10^−10^	β_2_	1.57 × 10^−3^	6.74 × 10^−5^
α_3_	9.99 × 10^−4^	8.18 × 10^−13^	β_3_	4.48 × 10^−5^	0.6561
α_4_	−3.32 × 10^−6^	1.19 × 10^−11^	β_4_	9.37 × 10^−7^	1.39 × 10^−29^
α_5_	4.23 × 10^−4^	0.0003	β_5_	1.32 × 10^−6^	1.88 × 10^−33^
α_6_	1.28 × 10^−4^	0.3651	β_6_	8.07 × 10^−5^	0.4686
α_7_	−7.35 × 10^−4^	0.3782	β_7_	2.17 × 10^−3^	0.0009
α_0_	−4.14 × 10^−2^	0.4342	β_0_	−1.45 × 10^−2^	0.0007
R^2^	0.78	4.56 × 10^−52^ **	R^2^	0.74	1.25 × 10^−40^ **

* Significance for a particular regression coefficient; ** for the whole layout.

**Table 9:** Regression function coefficients with R^2^ fitting for µ_m_ and T_µa_ models after verification.

µ_m_			T_µa_		
Coefficient	Value	Value F *	Coefficient	Value	Value F *
α_1_	2.76 × 10^−3^	3.79 × 10^−93^	β_1_	−2.81 × 10^−4^	7.85 × 10^−9^
α_2_	10.0 × 10^−4^	1.35 × 10^−12^	β_2_	1.53 × 10^−3^	0.0001
α_3_	4.26 × 10^−4^	0.0003	β_3_	9.46 × 10^−7^	3.23 × 10^−29^
α_4_	−3.32 × 10^−6^	1.49 × 10^−11^	β_4_	1.31 × 10^−6^	2.47 × 10^−32^
–			β_0_	−1.24 × 10^−1^	0.0039
R^2^	0.99	3.9 × 10^−235^ **	R^2^	0.71	5.54 × 10^−41^ **

* Significance for a particular regression coefficient; ** for the whole layout.

## Data Availability

The data presented in this study are available on request from the corresponding author.
